# Identification, Taxonomy and Performance Assessment of Type 1 and Type 2 Spin Bowling Deliveries with a Smart Cricket Ball

**DOI:** 10.3390/s23188012

**Published:** 2023-09-21

**Authors:** René E. D. Ferdinands, Batdelger Doljin, Franz Konstantin Fuss

**Affiliations:** 1 Discipline of Exercise and Sports Science, Sydney School of Health Sciences, Faculty of Medicine & Health, University of Sydney, Sydney, NSW 2141, Australia; 2 Smart Products Engineering Program, Swinburne University, Melbourne, VIC 3000, Australia; bdoljm@swin.edu.au; 3 Chair of Biomechanics, Faculty of Engineering Science, University of Bayreuth, D-95447 Bayreuth, Germany; franzkonstantin.fuss@uni-bayreuth.de; 4 Division of Biomechatronics, Fraunhofer Institute for Manufacturing Engineering and Automation IPA, D-95447 Bayreuth, Germany

**Keywords:** smart cricket ball, performance analysis, spin rate, spin bowling, wrist spin, finger spin, Type 1, Type 2, artificial intelligence

## Abstract

Spin bowling deliveries in cricket, finger spin and wrist spin, are usually (Type 1, T1) performed with forearm supination and pronation, respectively, but can also be executed with opposite movements (Type 2, T2), specifically forearm pronation and supination, respectively. The aim of this study is to identify the differences between T1 and T2 using an advanced smart cricket ball, as well as to assess the dynamics of T1 and T2. With the hand aligned to the ball’s coordinate system, the angular velocity vector, specifically the x-, y- and z-components of its unit vector and its yaw and pitch angles, were used to identify time windows where T1 and T2 deliveries were clearly separated. Such a window was found 0.44 s before the peak torque, and maximum separation was achieved when plotting the y-component against the z-component of the unit vector, or the yaw angle against the pitch angle. In terms of physical performance, T1 deliveries are easier to bowl than T2; in terms of skill performance, wrist spin deliveries are easier to bowl than finger spin. Because the smart ball allows differentiation between T1 and T2 deliveries, it is an ideal tool for talent identification and improving performance through more efficient training.

## 1. Introduction

Cricket is a sport that engages two main combatants: batters and bowlers. The bowlers are akin to the pitchers in baseball, attempting to get the batters “out” (or “dismiss” them) for the least number of runs. The bowlers form two distinct lineages: first, the fast bowlers releasing the ball at high speed, so as to reduce the time-window for the batter to perceive, react, and respond to the oncoming ball [1] and second, the spin bowlers imparting spin to the ball, so as to cause the ball to deviate in flight through the Magnus force and deviate off the pitch through friction [2], posing a more complex challenge to the batter, one in which the bat and body must move in response to an approximate prediction of changing flight and bounce trajectories. 

Each of the spin-bowling groups ramifies into the sub-lineages of wrist spin and finger spin [3]. Both these terms are misnomers based on historic usage rather than formal biomechanics since the fingers and wrist operate in each of these types of spin bowling. However, they label the two most basic methods of spinning the ball in different directions. For instance, a right-hand bowler delivering a finger spinner would cause the ball to deviate to the right (or leg side) after landing; whereas in the case of a wrist spinner, the ball would deviate to the left (or off side) [2,4]. The directions of ball deviation would be reversed for a left-handed bowler. 

Kinematic differences between these generic types of spin bowling have been found, which are presented subsequently. Beach et al. [3] found significant technical and performance differences between wrist spinners and finger spinners in the direction of spin angular velocity, approach speed, release height, and rear leg kinematics. In addition, wrist-spin bowlers tend to release the ball with a higher spin rate, a larger component of side-spin, and a lower absolute angle of spin axis elevation than finger spinners [3,5]. In addition, kinematic differences have been found between elite and non-elite spin bowlers. Spratford et al. [5] found that elite finger spinners bowled with significantly more spin rate and ball velocity than the development pathway finger spinners. The better performance of these elite finger spinners could be linked to a technique that includes a higher hip–shoulder separation angle, a mid-way pelvis angle and a relatively side-on shoulder angle at the front foot [6]. 

**Figure 1:**
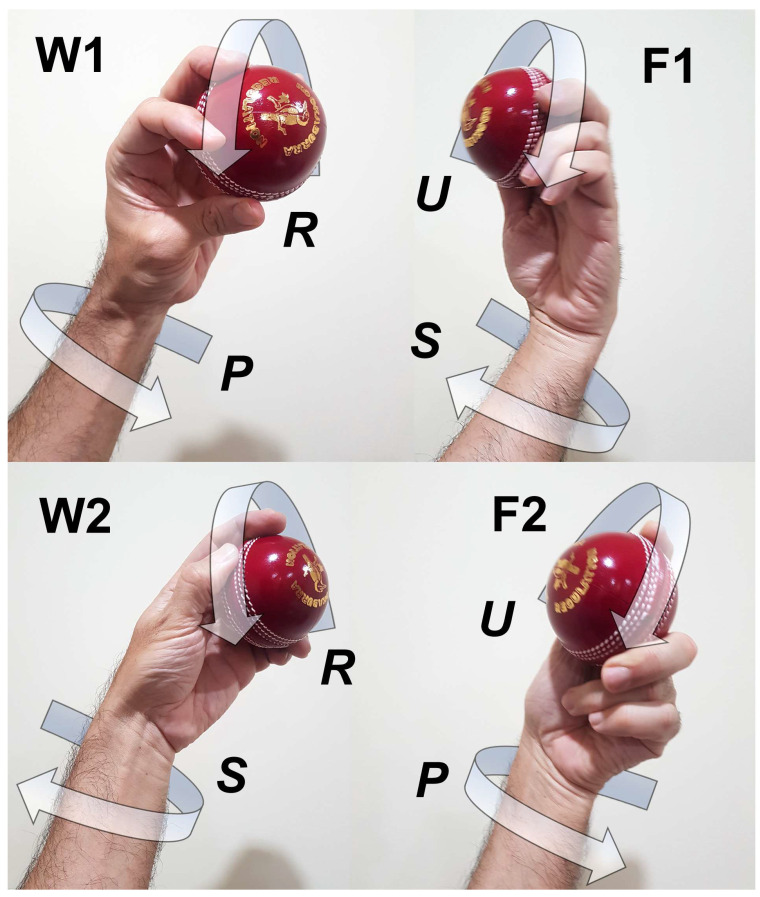
Directions of forearm and finger rotations when executing finger (F) spin and wrist (W) spin Type 1 and Type 2 deliveries; W1 = wrist spin type 1; F1 = finger spin type 1; W2 = wrist spin type 2; F2 = finger spin type 2; U = ulnar abduction of fingers; R = radial abduction of fingers; S = supination of forearm; P = pronation of forearm (cf. Table 1 and Figure 2).

**Figure 2:**
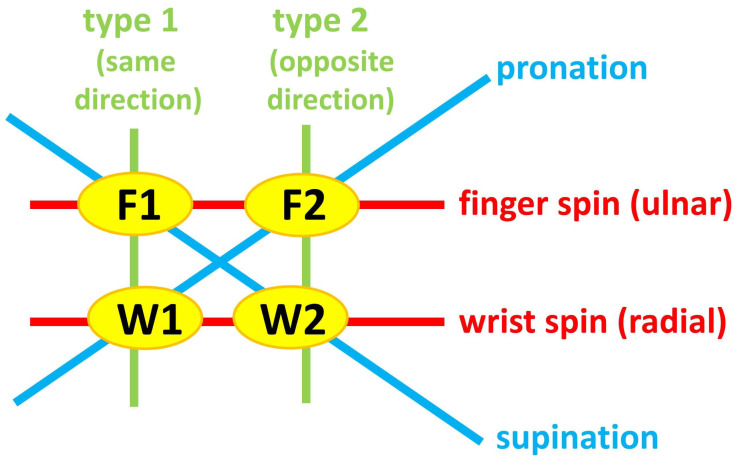
Schematic of the kinematics of forearm and finger movements when executing finger (F) spin and wrist (W) spin Type 1 (T1) and Type 2 (T2) deliveries; ulnar: left hand palmar view or right hand dorsal view, the fingers rotate the ball clockwise in ulnar direction; radial: left hand palmar view or right hand dorsal view, the fingers rotate the ball counterclockwise in radial direction; the terms “same direction” and “opposite direction” refer to the directions in which forearm and fingers move (cf. Table 1 and Figure 1); W1 = wrist spin type 1; F1 = finger spin type 1; W2 = wrist spin type 2; F2 = finger spin type 2.

From these early studies in spin bowling biomechanics, it has been established that technical and performance differences exist between wrist spin and finger spin. Hence, it is not considered effective coaching practice to specify one standard technical protocol for both wrist-spin and finger-spin bowlers [3]. Furthermore, in recent times, it has been found that each of wrist spin and finger spin can be produced through two distinct mechanisms. These mechanisms were first coined as Type 1 and Type 2 by Beach et al. [3], who found the existence of both types in a cohort of high-performance spin bowlers relative to their age. In Type 1 spin, both the forearm angular velocity and spin torque are in the same directional sense of rotation, whereas in Type 2 spin, they are in the opposite directional sense. Hence, Type 1 wrist-spin is bowled with forearm pronation and Type 2 wrist-spin with forearm supination: the process is the opposite for Type 1 and 2 finger-spin. (Table 1; Figure 1 and Figure 2). 

In the same manner that technical differences exist between the broad categorical types of leg spin and finger spin [3], technical differences could differentiate between Type 1 and Type 2 subtypes. The kinematics of the arm in athletic motions are dependent on motions remotely located in the kinetic chain, because a joint torque in a multi-segmental system can induce angular accelerations at all other joints in the system through dynamic coupling [7]. Hence, it is unlikely that spin bowlers can change the direction of forearm rotation independently of the other body segments, such as the thorax and pelvis that form part of the kinetic link chain. If this principle holds true then this implies that a Type 1 leg-spin bowler will require a different coaching scheme to that of a Type 2 leg-spin bowler, one that could differ in terms of body positioning, segmental sequencing, relative segmental planes of motion, and segment velocity contributions to spin rate. 

The discovery of these sub-types of spin bowling has been relatively recent, so spin bowlers have generally not been informed as to whether they supinated or pronated their forearms during the process of applying spin torque to the ball. The current method of identifying Type 1 and 2 spin bowling follows the traditional protocol: the testing of spin bowlers in a biomechanics laboratory with a marker-based motion analysis system operating at 200 Hz or higher. The basic process is to use a marker-based joint coordinate system to compare the directions of forearm rotation and spin angular velocity vectors to determine whether Type 1 or Type 2 spin has been bowled. 

Motion analysis is limited in its ability to assess these subtypes of spin. Firstly, the fingers are very difficult to track to quantify finger kinematics. The high number of markers required to define the motions of the finger segments would make it impractical: markers would be occluded during the bowling action and the excessive number of markers on the fingers would significantly impede performance. Secondly, the calculation of finger torque on the ball would be inaccurate. Finally, it would take a long time to process the data, rendering real-time feedback of performance virtually impossible.

With these limitations, lab-based motion analysis does not offer a practical means of establishing a cohesive taxonomy of spin bowling types. Apart from inhibiting spin bowling performance, the technology simply cannot generate the required number of critical kinematic and kinetic variables to classify the major mechanisms of spin bowling. However, another means of instrumental analysis can be used that is even suitable for the playing field: an advanced smart cricket ball has been developed [8,9,10,11,12] that can calculate four physical parameters (resultant torque, spin torque, power, and angular acceleration) and five skill parameters (precession, normalised precession, precession torque, efficiency, and ratio of angular acceleration to spin rate), while measuring the spin angular velocity at 815 Hz [10,11,12]. Armed with this diverse assortment of variables, the biomechanist is better equipped to formulate a hierarchical taxonomy of spin bowling in which deliveries are organized into groups or types based on biomechanical parameters. 

Taxonomies are commonly developed in sports to classify techniques according to common characteristics. In tennis, the standard taxonomy of grips includes the continental, Eastern, semi-Western, and Western, listed in order of increasing supination for the forehand, and conversely in the order of increasing pronation for the backhand. Several injury types have been associated with this taxonomy, including radial side injuries in the Eastern grip, and ulnar side injuries in the semi-Western and Western grips [13]. The notable aspect here is that the more widely separated the grips in terms of taxonomy, the greater the difference in corresponding techniques. A more striking example can be found in table tennis between players who use the conventional “shake hands” grip and the “pen-holder” grip [14,15,16]: the technical demands of the forehand and the backhand differ considerably, even resulting in contrasting strategic processes of play. If the mere method of holding the end-effector in sports can affect the entire execution of the stroke or shot, then it should be expected that differences between Type 1 and Type 2 spin, which are defined by dynamic differences, would likewise result in technical differences in their execution. It then becomes of paramount importance to identify these sub-types of spin so that coaches can match the correct technical models with Type 1 and Type 2 spin bowlers (wrist spin or finger spin). 

The aim of this study is to assess whether an advanced smart cricket ball can identify the differences between Type 1 and Type 2 spin based on kinematic, kinetic and dynamic variables that are difficult, and in some cases impossible, to measure using conventional motion analysis. The hypothesis is that the smart cricket ball can explore the inter-relationships in the variables that differentiate between the sub-types of spin that will assist in the development of a taxonomic spin bowling classification based on biomechanical parameters. It is envisaged that such a taxonomy will assist biomechanists to identify the relationship between Type 1 and Type 2 spin, providing a basis for coaches to prescribe type-matched techniques and sets of variations. 

## 2. Materials and Methods

### 2.1. The Smart Ball

The smart cricket ball was developed in late 2011 [8,9], and is a typical example of instrumented or ‘smart’ sports equipment [17]. The electronics (printed circuitry board, battery) were miniaturized, and wireless data transfer and charging was implemented in 2014 [10]. The ball is instrumented with 3 high-speed gyros that are aligned orthogonally and measure the spin rate (angular velocity *ω*) of the ball at 815 Hz [12]. The raw *ω* data are filtered with a Butterworth low-pass filter of the 3rd order with a cut-off frequency of 30 Hz [18,19,20]. The smart ball is of legal mass and fully balanced. The ball is controlled (switch on/off, data download) via laptop or smartphone. The smart ball provides 10 performance parameters, 5 physical and 5 skill parameters. From the measured *ω*, the angular acceleration *α* is calculated from *ω*’s 1st time derivative. From Euler’s equations (and *ω* and *α*) the resultant torque *T_R_* is determined. The **T_R_** vector has two components: the spin torque **T_s_**, which is parallel to, and changes the magnitude of, **ω**; and the precession torque **T_p_**, which rotates the **ω** vector into the **T_R_** vector with respect to the ball [12]. Finally, the power *P* is calculated from *ω* and *T_s_*. In terms of skill parameters, in addition to *T_p_*, the precession *p* is calculated from *T_p_* and *ω*, and corresponds to the angular velocity of the moving spin rate vector **ω**. The normalised precession *p_n_* denotes the angle between **T_R_** and **ω** vectors. The efficiency *η* is the ratio of the actual rotational energy of the ball to the ideal energy, if *T_R_* = *T_s_*, and *T_p_* = 0. The frequency (*α*/*ω*) is the ratio of maximum *α* to maximum *ω*, which explains how efficiently *α* increases *ω*. From a coaching perspective, the physical performance parameters (*ω*, *α*, *T_R_*, *T_s_*, *P*) have to be maximized, whereas the skill parameters *T_p_*, *p*, *p_n_* and *α*/*ω* have to be minimised and *η* to be maximized [12].

### 2.2. Participant

The participant, a retired first-class cricketer and the first author of this paper, is proficient in bowling a wide range of deliveries, including topspin, sidespin, and backspin, all of them for both finger and wrist spin. Only one participant was found who had demonstrated the ability to bowl both Type 1 and Type 2 sub-variants of finger spin and wrist spin, an ability based on years of bowling and demonstrating these deliveries at high-level competition and coaching at international levels, respectively. The participant was therefore able to bowl all 4 deliveries, finger spin types 1 and 2 (F1, F2), and wrist spin types 1 and 2 (W1 and W2) at the same perceived performance level. All bowling trials were performed indoors in dry conditions. If any moisture appeared on the ball, the participant cleaned the ball on his trousers, as in normal playing conditions. This study was granted ethics approval by the Swinburne University Human Ethics Committee (approval no. 20191582-3216) and adhered to the Declaration of Helsinki.

### 2.3. Experiments

The participant bowled the 4 deliveries identified in Table 1 and Figure 1 and Figure 2 (all of them are sidespin deliveries) 6 times each with the smart cricket ball. The ball had to be held such that the *z*-axis of ball’s sensor coordinate system points out of the palm in left handers, and into the palm in right handers; and index finger is placed at intersection of the positive *x*-axis and the seam. This convention allows distinguishing between finger and wrist spin, as the z-component of the angular velocity vector is positive in wrist spin after release, and negative in finger spin.

### 2.4. Data Analysis 1—Performance Assessment

The raw data provided by the smart cricket ball were processed with the smart cricket ball software [11]. The software calculates the vector diagram of the angular velocity as well as the performance parameters [11]. To detect differences in performance across the 4 deliveries, all 10 performance parameters were compared in pairs (F1 and F2, W1 and W2, F1 and W1, F2 and W2, F2 and W1, F1 and W2) with the Mann–Whitney U test, and the p-values and effect sizes (r = 1–2 U n_1_^−1^ n_2_^−1^) were calculated. The effect sizes were interpreted according to McGrath and Meyer [21]. Note that the performance parameters do not necessarily reflect the performance of a bowler, but also express the inherent dynamics of individual deliveries. According to Fuss et al. [12], topspin deliveries are more efficient than backspin deliveries, and wrist-spin deliveries are more efficient than finger-spin deliveries. Subsequently, each performance parameter was normalised to its rank within the data range across all 4 deliveries, where higher performance corresponds to higher ranks. The normalised data of both performance groups (physical and skill) were compared pairwise with the Mann–Whitney U test.

### 2.5. Data Analysis 2—Clustering of the 4 Deliveries

**Figure 3:**
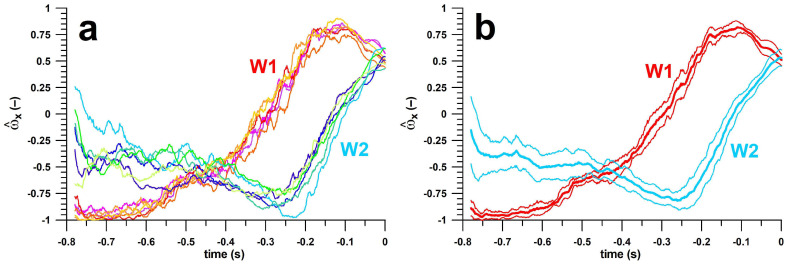
x-component of the unit vector of the angular velocity (ω) vs. time; (**a**): raw data (6 datasets per delivery); (**b**): average ± 1 standard deviation; W1 = wrist spin type 1, W2 = wrist spin type 2.

For separating all 4 deliveries, we used the angular velocity (*ω*) data provided by the smart ball software (Figure 3a). To align the data dynamically, we set the timestamp at the torque peak to t = 0. We distinguished the T1 and T2 deliveries from the relative magnitude of **ω** (x,y,z-components of unit vector), and its direction (Euler angles, i.e., yaw and pitch) in the ball coordinate system (xy-plane = plane of the seam). 

We verified the continued separation of Type 1 and 2 data from the included angle between angular velocity unit vectors, calculated from the inverse cosine of their dot product, of finger spin Type 1 and 2, and wrist spin Type 1 and 2.

At each timestamp, we determined the average and the standard deviation of each parameter (e.g., x-component of the **ω**-unit vector of wrist spin Type 1; Figure 3b), as well as the p-value of the corresponding Type 1 and 2 data. The p-value served for identifying the periods during which the Type 1 and 2 data are not significantly different, and thus cannot be used for distinguishing between the 2 types. The 5 parameters (**ω_x_**, **ω_y_**, **ω_z_**, yaw angle, pitch angle) were subsequently reduced to 4 by excluding one not suitable for clearly separating the 2 types. 

Subsequently, we plotted the averages of the 4 deliveries identified in Table 1 and Figure 1 for each parameter, and unveiled common and uncommon behaviour, suitable for distinguishing between Type 1 and 2 deliveries. The best timestamp for maximally separating the deliveries’ parameters resulted from the maximum of the p-values’ average, or, more precise, from the average of –log_10_(p), under the condition that all 8 p-values must be smaller than 0.05. The 8 p-values result from the comparison of type 1 and 2 for finger spin and wrist spin, in terms of **ω_y_**, **ω_z_**, yaw, and pitch).

The method described herein represents the first step for the development and training of an artificial intelligence model for automated decision making and detection of Type 1 and 2 deliveries.

## 3. Results

The results section is organized in the following way. The subsection *Performance Analysis* establishes the similarities and differences between the four deliveries, and shows that the four deliveries are individual and separate entities. The differences are shown by means of the kinematic parameters (movement of the spin axis, **ω**-vector, with respect to the ball), and 10 performance parameters (5 physical and 5 skill parameters). The performance parameters provide information about which delivery is more efficient when bowling or produces a higher spin rate.

The second subsection, *Clustering of the data of each delivery*, provides the foundation for an artificial intelligence algorithm that selects the kinematic parameters (x,y,z unit vectors of **ω**, and its yaw and pitch angles) and the right point in time to achieve maximal separation of the four deliveries on a two-dimensional map.

### 3.1. Performance Analysis

**Figure 4:**
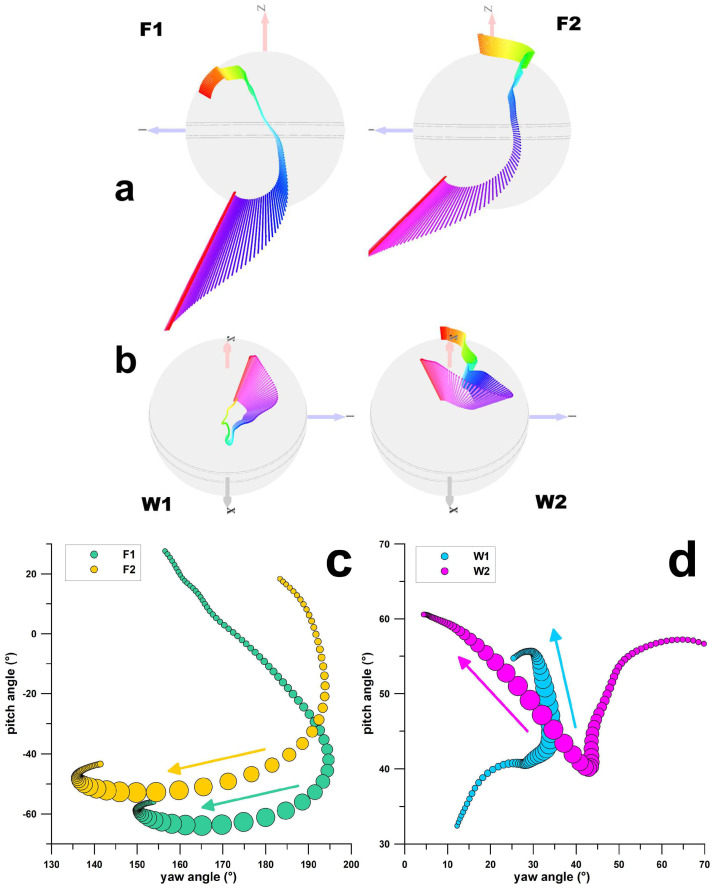
(**a**,**b**): 4D vector diagrams of the angular velocity (**ω**) up to the release of the ball; the length of **ω**-vectors corresponds to the magnitude of ω, time is colour-coded (4th dimension); (**c**,**d**) plate carrée map projection of the pitch angle against the yaw angle of the **ω**-vectors; the bubble size denotes the magnitude of the torque imparted on the ball; F1 = finger spin type 1; F2 = finger spin type 2; W1 = wrist spin type 1; W2 = wrist spin type 2.

The average angular velocity vector diagrams of all four deliveries are shown in Figure 4a. To understand the differences of the rotating ball when executing the deliveries, the intersection points of the consecutive vectors with the surface of the ball, i.e., the paths of the spin axis, are shown by means of a plate carrée map projection in Figure 4b,c. F1 and F2 deliveries exhibit similar trends and shapes of the spin axis paths, whereas W1 and W2 deliveries are different.

Table 2 shows that the performance data of individual pairs were completely separated (not overlapping) in 58%. Across the 10 performance parameters and 6 pairs per performance parameter, W1 proved to be superior in terms of performance over the other three deliveries, followed by F1 and W2 with similar performance, and finally by F2 with the lowest performance. In terms of physical performance, W1 had a higher performance, followed by F1; F2 and W2 had the lowest and similar performances (Table 3). In terms of skill performance, W1 led, followed by W2, F1 and finally F2.

**Figure 5:**
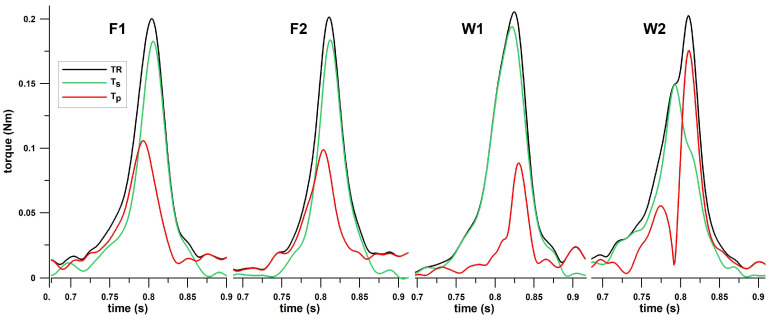
Torques against time (for comparative reasons, deliveries with approximately the same TR magnitude are shown here); F1 = finger spin type 1; F2 = finger spin type 2; W1 = wrist spin type 1; W2 = wrist spin type 2; TR = resultant torque; Ts = spin torque; Tp = precession torque.

The torque diagrams are shown in Figure 5. There is no difference between F1 and F2 in terms of shape and magnitudes of *T_R_*, *T_s_* and *T_p_*, which matches the results of Figure 4b. The *T_p_* spike is located in the first half of the *T_R_* spike. The difference between W1 and W2 is obvious from the *T_p_* data: a single *T_p_* spike in the second half of *T_R_* for W1, and two *T_p_* spikes with the second one being excessively high for W2. Equally, this striking difference matches the results of Figure 4c. 

### 3.2. Clustering of the Data of Each Delivery

**Figure 6:**
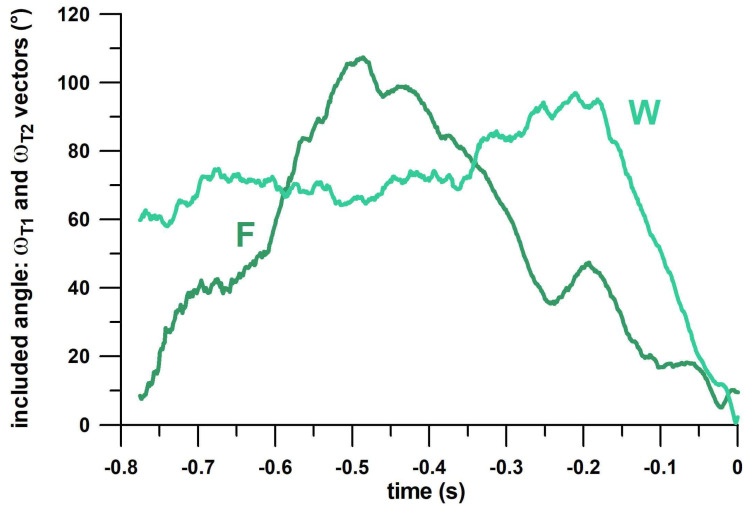
Angle between the angular velocity (ω) vectors of Type 1 (T1) and Type 2 (T2) plotted against time, for finger spin (F) and wrist spin (W) separately.

The kinematics of Type 1 and 2 spin-bowling data are clearly different (Figure 6). The angular velocity vectors of Type 1/2 finger-spin deliveries are separated from 0.8 s before the torque peak up to the torque peak (t = 0 s) by an included angle between 0° (at –0.8 s and 0 s) and 107° (at approximately –0.5 s) on average (Figure 6). The included angle between angular velocity vectors of type 1 and 2 finger spin and type 1 and 2 wrist spin is greater than 40° for a substantial portion of time prior to release, clearly demonstrating the differences between the Type 1 and Type 2 deliveries. The angular velocity vectors of Type 1/2 wrist-spin deliveries are already separated earlier than 0.8 s before the torque peak up to the torque peak (t = 0 s) by an angle between 0° (at 0 s) and 97° (at approximately –0.2 s) on average (Figure 6).

**Figure 7:**
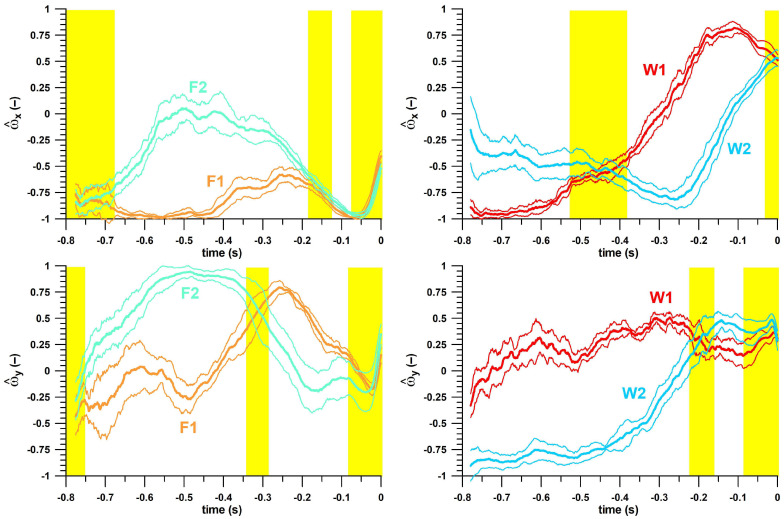

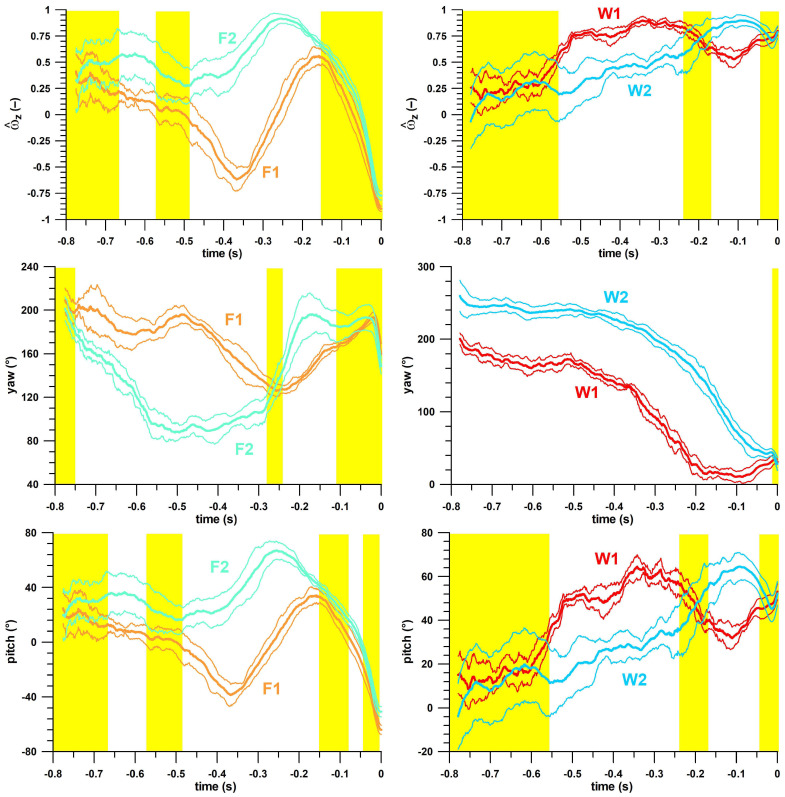
Kinematic parameters against time for finger spin and wrist spin Type 1 and 2 deliveries (average ± 1 standard deviation); the kinematic parameters are: x-, y-, and z-components of the unit vector of the angular velocity (ω), and Euler angles (yaw, pitch) of the unit vector; F1 = finger spin type 1, F2 = finger spin type 2; W1 = wrist spin type 1, W2 = wrist spin type 2; the yellow zones indicate time zones unsuitable for distinguishing between Type 1 and Type 2 deliveries.

As an overall differentiation between deliveries, all deliveries can be categorised into specific zones according to their angular velocity components and pitch and yaw angles. The five individual kinematic parameters (x-, y-, and z-components of the unit vector of the angular velocity **ω**, and Euler angles [pitch, yaw] of the unit vector; Figure 7) are partially overlapping as shown by the yellow zones in Figure 7.

**Figure 8:**
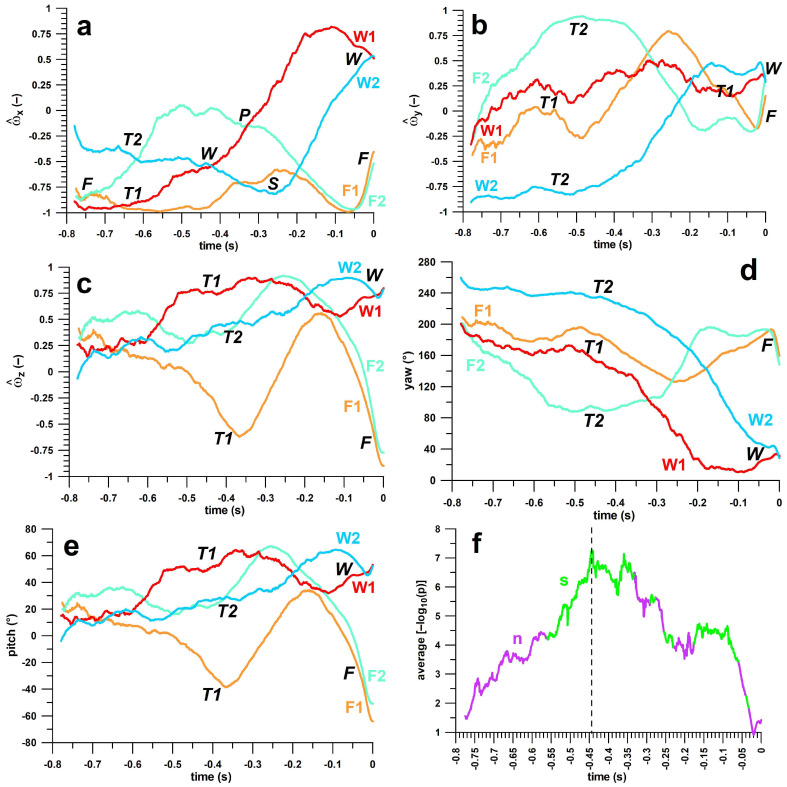
(**a**–**e**): kinematic parameters (components and Euler angles of unit vector ω) against time, for identification of common and uncommon features of the averages of finger(F) spin and wrist(W) spin Type 1 (T1) and Type 2 (T2) deliveries; F1 = finger spin type 1, F2 = finger spin type 2; W1 = wrist spin type 1, W2 = wrist spin type 2; P = pronation, S = supination; (**f**) differentiation between T1 and T2 from critical time stamps at which T1 and T2 are maximally separated (8 parameters: ω_y_, ω_z_, yaw, and pitch of finger spin and wrist spin); p = p-value; log_10_ = decadic logarithm; s (green) = significant: all 8 p-values < 0.05; n (purple) = not significant: at least one p-value > 0.05.

When considering the averages of the five individual kinematic parameters of all four deliveries (Figure 8a–e), we can distinguish between common and opposite trends. For example, the unit vectors of *ω_y_* (Figure 8b) show common trends for Type 1, whereas the trends of Type 2 deliveries are not comparable. At *t* ≈ –0.5 s, the unit vector of *ω_y_* is 0 ± ¼ for Type 1, whereas for Type 2, the *ω_y_* values for finger and wrist spin are extreme, greater than +¾ and smaller than –¾, respectively. The unit vectors of *ω_y_* (Figure 8b) and the yaw angles (Figure 8d) of the unit vectors show the same behaviour before *t* = –0.3 s for Type 1. The same applies to the unit vectors of *ω_z_* (Figure 8c) and the pitch angles (Figure 8e) of the unit vectors before *t* = –0.3 s for Type 2. For the unit vectors of *ω_x_* (Figure 8a), common features are only local: finger spin at –0.75–−0.7; Type 1 at –0.65; Type 2 at –0.65–−0.6; wrist spin at –0.45–−0.4; pronation at –0.35–−0.3; and supination at –0.35–−0.2. The unit vector of *ω_x_* is therefore not suitable for clearly distinguishing between Type 1 and Type 2, and we therefore excluded *ω_x_* from further analysis. 

**Figure 9:**
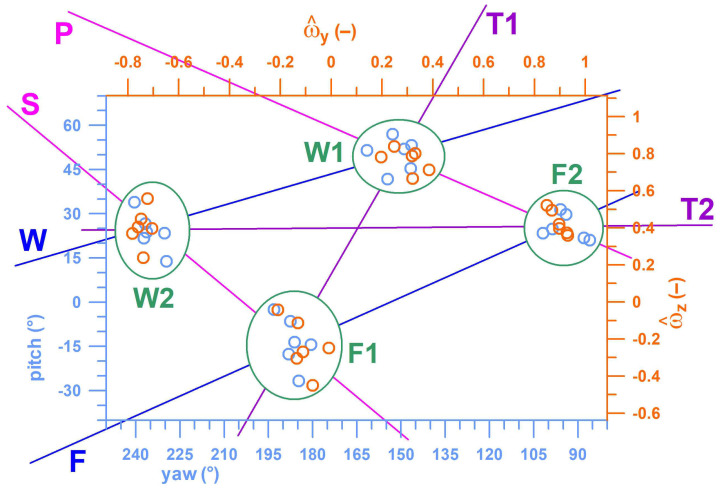
Pitch angle vs. yaw angle of the angular velocity vector (of the smart ball) and z-component vs. y-component of the unit vector (^) of the angular velocity (ω); the 4 data clusters (taken at 0.44 s before the peak of the torque spike) are identified by 4 ellipses; W = wrist spin, F = finger spin; T1 = type 1, T2 = type 2; F1 = finger spin type 1, F2 = finger spin type 2, W1 = wrist spin type 1, W2 = wrist spin type 2; P = pronation, S = supination.

From the remaining four individual kinematic parameters (*ω_y_*, *ω_z_*, yaw, pitch), the timestamp of the maximum separation of all four deliveries was determined from the average of –log_10_(p), and the maximum average separation of deliveries was found at *t* = –0.44 s (cf. dashed line in Figure 8f). Table 4 shows the R^2^-values of the correlations between Euler angles (yaw, pitch) and unit vectors (*ω_y_*, *ω_z_*) of the angular velocity. The yaw angle correlated with *ω_y_*, and the pitch angle with *ω_z_*. *ω_x_*, however, was not significantly correlated. The corresponding linear regression functions served for aligning *ω_y_* to the yaw angle, and *ω_z_* to the pitch angle in Figure 9. Figure 9 shows the four clusters of the four deliveries clearly separated at *t* = –0.44 s. This cluster diagram also shows the separation of finger- and wrist-spin deliveries, as well as supination and pronation, comparable to the diagram in Figure 2. 

**Figure 10:**
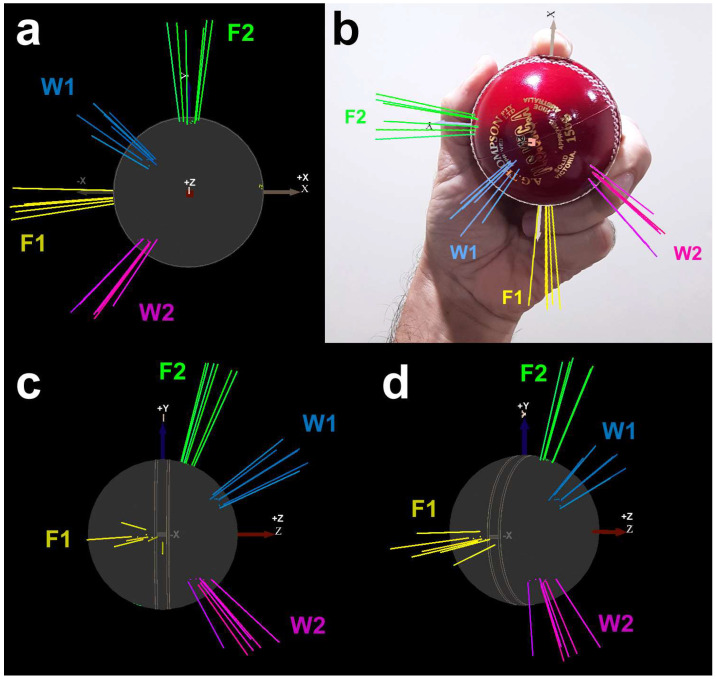
3D vector diagram of the unit vectors identified as clusters in Figure 9; each line represents the 3D unit angular velocity vector for a single delivery; there are 6 lines per cluster, demarcating the territories of the spin delivery types; the angular velocity vectors are shown on the cricket ball in pole view (**a**), with the same view projected on a real cricket ball held by a left hand (**b**); in seam view (**c**); and in isometric projection (**d**); F1 = finger spin type 1, F2 = finger spin type 2, W1 = wrist spin type 1, W2 = wrist spin type 2; the grip in subfigure (**b**) is a neutral one, applicable to all 4 deliveries (F1, F2, W1, W2), and not a grip at 0.44 s before the peak torque (approximately 0.6 s before release), as these grips are different (cf. Figure 1).

Figure 10 shows the 3D vector diagrams of the unit vector (*ω_x_*, *ω_y_*, *ω_z_*) data featured as clusters in Figure 9. The same clusters apply, proving that the four deliveries are different and separated entities.

## 4. Discussion

Spin bowling is arguably the most complex and technically diverse of the bowling genres in cricket. Through a process of technical experimentation, spin bowlers have been able to generate a wide array of spin bowling deliveries, each of which induces a different combination of aerodynamical and rebound effects on the ball, for the purpose of confounding the batter’s perception to increase the chances of dismissal. From the earliest days of cricket, four basic directions of spin bowling were recognised, namely, off spin, leg spin, topspin, and backspin (often referred to as “check-spin” in the early coaching literature). Beldam and Fry [22] illustrated that a combination of topspin and sidespin was a more practical form of delivery than “pure” sidespin, an early suggestion that hybrid forms of spin bowling deliveries were possible. As cricket evolved, more deliveries were added, including the googly, flipper, carrom ball, and doosra. All these deliveries were loosely grouped under the categories of either wrist spin or finger spin. Although these categories have been universally adopted by the cricket fraternity and are useful in terms of demarcating basic genres of spin bowling, a formal taxonomic system of classification of spin bowling deliveries based on strict anatomical and biomechanical criteria has not yet been developed. Moreover, a formal taxonomy is especially needed now that Type 1 and Type 2 spin-generating mechanisms have been discovered in both wrist-spin and finger-spin bowling [3].

### 4.1. Smart Ball Validation of Spin Bowling Deliveries

The objective of devising a formal taxonomy in spin bowling is to classify spin bowling deliveries into sets or types based on their shared traits and lineage. A scientific-based taxonomy considers the functional and logical relationships between variables as criteria for grouping in sets. Such an endeavour is predicated on the ability to collect a range of relevant biomechanical data on spin bowling performance. In this paper, it is proposed that the smart cricket ball is particularly effective for this purpose. The smart cricket ball is an instrumented cricket ball [17] that can measure various kinematic and kinetic performance variables, including variables that cannot be measured accurately by a motion analysis system. Furthermore, the measurement does not require the external placement of markers on the fingers, minimising any interference with performance. 

An initial indication of differentiation between spin bowling deliveries is apparent through qualitative examination of the 4D-vector diagrams of angular velocity (Figure 4a). The angular velocity vector traverses along the ball differently for finger spin and wrist spin. Then a plate carrée map projection of the pitch angle against the yaw angle shows that the F1 and F2 deliveries mapped similarly; however, the maps of W1 and W2 deliveries were divergent in both path and magnitude characteristics (Figure 4b,c). The importance of this cursory examination of spin angular velocity maps should not be undervalued. It demonstrates that the smart ball could detect significant differences between spin bowling deliveries, not merely between the traditional categories of finger spin and wrist spin, but at a more refined level for wrist spin, between the sub-types of Type 1 and Type 2. Furthermore, it is the angular velocity vector that distinguishes between these sub-types. Working on the basis that kinematic differences serve as a means of differentiating between spin bowling deliveries, it follows that a taxonomic classification of spin bowling with smart ball data may be feasible, and this encourages a more intensive analysis, including kinetics. 

Kinetics has the advantage over kinematics in that it quantifies mechanisms at a more causal level of analysis. A qualitative observation of torque-time graphs shows distinct variations between the spin and precession torques (Figure 5). Gross differences are seen in the torque-time histories between finger spin and wrist spin: both T1 and T2 finger spin show the peak precession torque occurring before the peak spin torque, whereas the timing of these peak torques is reversed in the case of wrist spin. A further differentiation is clearly apparent between T1 and T2 wrist spin, with the T2 spin torque being double-peaked and of a much higher magnitude than T1 (Figure 5). Particular attention should be drawn to the T2 wrist-spin precession torque, which is approximately 1.5 times higher than the other deliveries. Precession torque can be considered an “unwanted” torque, and hence the inverse of its value is a measure of spin-generation efficiency. By this measure, T2 wrist spin is not only clearly differentiated from the rest of the spin bowling deliveries but is a particularly mechanically inefficient mode or technique of generating spin [12]. 

From the analyses conducted thus far, the smart ball can differentiate between finger spin and wrist spin, and between top-, side-, and backspin. However, differentiation between Type 1 and Type 2 is more effectively observed through the included angle between the angular velocity vectors of F1 and F2, and W1 and W2 (Figure 6). For instance, W1 and W2 angular velocities have an included angle of 60 degrees or higher until about 0.1 s before ball release. F1 and F2 have more a restricted range of included angle values above this threshold, from −0.6 to −0.3s, but this included angle reaches even higher values, higher than 90 degrees during the mid-portion of this period. This preliminary data suggests that the included angle can differentiate between Type 1 and Type 2 sub-types, justifying a more detailed analysis of the variables related to the angular velocity vector. Hence, a graphical comparison of type 1 and 2 for finger spin and wrist spin in terms of five individual kinematic parameters (x-, y-, and z-components of the unit vector of the angular velocity *ω*, and Euler angles (pitch, yaw) of the unit vector) clearly showed differentiation in several specific non-yellow zones (Figure 7). 

By observing the trends in the averages of the five individual kinematic parameters of all four deliveries (Figure 8a–e), it seemed unlikely that *ω_x_* would be suitable for distinguishing between Type 1 and Type 2; only local trends are observable. In addition, correlation analysis found that *ω_y_* was correlated with yaw and *ω_z_* was correlated with pitch, but ωx was uncorrelated (Table 4); and being unable to constitute a functional relationship with other variables, it was removed from further analysis. From the remaining four individual kinematic parameters (*ω_y_*, *ω_z_*, yaw, pitch), the timestamp of the maximum average separation of all four deliveries was found at *t* = –0.44 s (Figure 8f). Then, at this time point, linear regression functions were calculated and plotted in a way that aligns *ω_y_* to the yaw angle, and *ω_z_* to the pitch angle (Figure 9), resulting in four separated clusters, their boundaries clearly demarcated. This cluster diagram is means of validating different types of deliveries—in this case, showing that the bowler uses distinct mechanical processes to execute F1, F2. W1, and W2, supporting the qualitative schematic of the kinematics of forearm and finger movements in Figure 2.

The mechanical differences between these types of spin bowling deliveries are most notably observed on the 3D vector diagram of unit vectors on the ball, in which the grip shown is a neutral one, applicable to all bowlers (Figure 10). In this diagram, each line represents the 3D unit angular velocity vector for a single delivery. Hence, the six lines per cluster demarcate the discrete territories of the spin bowling deliveries, implying the generation of different spin-torque for each delivery type. In other words, the fingers can apply spin to the ball in patterns that correspond to different delivery types. The coaching literature has not explored this aspect of spin bowling technique. The smart ball potentially reveals the hidden layer of technique that underlies the subtle differences between spin bowling deliveries, in particular the differences between T1 and T2, for which no theoretical framework currently exists.

Further separation of the spin bowling deliveries (F1, F2, W1, W2) could be determined from the Mann–Whitney U test of 10 performance factors, with separation between the deliveries in 58 cases (Table 3). These variables could be combined in a way to provide more meaning, as physical and skill performance factors, ending up in different rankings of effectiveness for each of these types. This shows that the separation is clearly functional, separating deliveries in terms of real performance outcomes and also between the efficiency of delivering each ball, suggesting that these categories are real, affecting when they are used, having different levels of effectiveness, and being amenable to particular styles and strategies.

### 4.2. Coaching Implications

The smart ball was shown to differentiate between finger-spin and wrist-spin deliveries. However, coaches could dismiss this achievement, claiming that the technical differences between these classic types of spin bowling are easily discernible, even during live play. However, the smart ball could also identify Type 1 and Type 2 spin bowling deliveries without the use of a motion analysis system. This is an impressive accomplishment because these categories of spin bowling have only recently been discovered, and their distinctions can be difficult for humans to notice in real time. Although the pitch, yaw angle and angular velocity of a cricket ball are theoretically measurable by 3D-motion analysis, this would require intruding upon the performance within a biomechanics laboratory, which does not constitute an environmental setting that mimics real-world conditions. Furthermore, the smart ball has shown significant differences between F1, F2, W1, and W2 spin bowling deliveries on a cluster diagram of pitch angle vs. yaw angle of the angular velocity vector (of the smart ball) and z-component vs. y-component of the unit angular velocity vector (Figure 9). These four different types of spin require a varying number of mechanical motions to be executed correctly. As such, coaching requirements may differ depending on which delivery type a spin bowler wants to learn. Each of these sub-types of spin deliveries is unique: this is evident by observing the relative equidistant separation of their clusters (Figure 9) 

To further understand the coaching implications with further precision and effectiveness, it is advantageous to categorise the smart ball data into physical performance factors, skill performance factors, and technical and strategic applications. By taking the time to interpret the smart ball data under these categories, coaches and trainers can gain a more comprehensive understanding of spin bowling and be better equipped to help their players reach their full potential.

#### 4.2.1. Physical Performance Factors

The smart ball has been proven to be able to distinguish between performance outcomes, which include spin rate and other closely related variables (Table 2). Spin rate is related to torque, power, and angular acceleration. These results generally indicate better performance and can be analysed at either the individual (intra-subject) or collective (inter-subject) levels. Intra-subject comparisons involve measuring how well the subject performs each delivery. Hence, it is possible to conduct intra-delivery studies where one spinner’s spin rate is compared across multiple deliveries, establishing a performance rank of each variation of delivery. Inter-subject analyses compare different bowlers’ ability to perform these deliveries, a means of establishing baseline performance levels of spin bowlers or serving as a tool for talent identification. In addition, physical performance factors can also indicate performance trends between deliveries. For instance, for the spin bowler in this study, the W1 spin rate was higher than W2. If in subsequent studies with multiple subjects, this result was also found, then it can be concluded that W1 and W2 differ in the property of spin rate.

It is interesting that the physical performance factors were W1, F1, F2, W2 in order of decreasing performance. It was expected that W1 would be placed at the top of this list because wrist spin is known to generate a higher spin rate than the other types of delivery. Furthermore, Type 1 wrist spin, which is W1, is the conventional technique for wrist spin, in which the bowler pronates the forearm while imparting spin to the ball. From a conventional perspective, F1 would be considered the next best-performing spin delivery, because it is the conventional technique of finger-spin bowling, requiring the supination of the forearm while the fingers impart spin to the ball. Type 2 spin deliveries have been found in the laboratory but have not been yet implemented in the published coaching literature. Hence, coaches could be surprised that the Type 2 finger spin deliveries performed the Type 2 wrist-spin deliveries. 

These physical performance factors bring up important implications for coaches. Coaches need to be aware of the physical performance factors that influence spin bowling outcomes. The spin rate generated by the T1 wrist-spin delivery is substantially higher in comparison to both types of finger-spin delivery. Hence, it is important that wrist spinners are given focused attention and specialised coaching in spin bowling development squads to ensure they attain their full potential. In particular, specialised technical knowledge is required to teach wrist-spin bowlers to optimise their techniques to bowl T1 deliveries. In addition, the bowling action biomechanics of wrist spin differ considerably from finger spin, implying that coaches adjust their coaching strategies according to the genre of spin bowling [3]. Furthermore, coaches are faced with the challenge of upgrading their knowledge to identify spin bowlers who bowl the F2 and W2 spin deliveries. These spin deliveries can increase the level of deception of a bowler, as the spin torque applied to the ball is opposite to the direction of the forearm rotation. As such, the coach must be able to identify the spinners who employ these deliveries and understand exactly how these balls can be executed as efficiently as possible, recognising that they will not generally perform as their Type 1 counterparts do. 

Ultimately, this research regarding the physical performance factors of spin deliveries provides valuable technical insight for coaches when looking to maximise the performance of their bowlers. Even through the qualitative observation of plate-carée maps (Figure 4) and four-dimensional plots of pitch–yaw angular velocity components (Figure 9) generated by the smart cricket ball, coaches and researchers will find a more effective means of discerning and sorting spin bowling deliveries into their respective technical categories for a better analysis.

A closer look at the skill performance factors may provide insight into the reasons why F2 outperforms W2, as wrist-spin performance outcomes are generally higher than for finger spin, most notably the spin rate.

#### 4.2.2. Skill Performance

A player’s skill performance is determined by the amount of precession, which is a key factor in evaluating the quality of efficiency of the spin-generation mechanism, which is influenced by how the fingers interact with the ball. When a bowler generates spin torque with lower precession, the delivery will be more efficient since precession prevents the generation of energy. In such a case, more spin torque can be applied to the ball, causing a higher spin angular acceleration, which will lead to a higher spin rate, the primary performance outcome. Hence, skill performance factors are at the core of most coaching applications. As precession values are calculated per delivery type, coaches can gain a better understanding of which bowlers can deliver the ball with a more efficient technique. Benchmarking players by establishing a baseline performance is a standard coaching practice. Once this benchmarking has been completed, the coach can use high-speed motion to observe bowling technique more closely and devise technical interventions where appropriate. Afterwards, the coach can figure out which balls should constitute a bowler’s delivery set or what balls could be improved based on how effective each type of delivery is.

The spin bowler in this study spun the deliveries in the following skill performance order: W1, W2, F1, and F2 (Table 2 and Table 3). Firstly, this shows that in general, his wrist-spin bowling was more efficient than his finger-spin bowling; secondly, that the Type 1 variants within wrist-spin and finger-spin bowling were more efficient than their Type 2 counterparts. Precession, normalized precession, and precession torque are the major determinants of calculating skill performance order. Hence, precession could differentiate between these main types of spin bowling. From this perspective, Type 1 deliveries are more efficient than Type 2 deliveries because they minimize the amount of energy needed to spin the ball. Since the fingers and forearms tend to rotate in opposite directions during a Type 2 delivery, they end up creating extra precession torque, which requires additional energy to overcome. In contrast, Type 1 deliveries are much simpler and thus require less energy to complete because it is much easier to rotate the fingers and forearm in the same rotational sense during the imparting of spin to the ball. Hence, W1 was found to be most efficient in terms of skill performance, followed by W2, F1 and finally F2 (Table 3). 

As a further note, for a spin bowler, it is imperative to analyse both the skill performance order as well as the physical performance order of their deliveries. In terms of analysing both of these lists, W1 is the most desirable delivery type since it is both the most effective and efficient. However, even though W2 is the least efficient spin delivery, it is the second-highest-performing one (Table 3). This is a sign that even though W2 is relatively inefficient, one can still spend a large amount of energy to achieve a relatively high-performing spin delivery. In other words, W2 should not necessarily be treated as an inferior type of delivery to W1. Ultimately, the choice of which type of delivery to use is up to the individual bowler: bowlers should experiment with both types of deliveries to determine which one works best for them. With the right technique, W2 can be a highly effective delivery type.

#### 4.2.3. Technical and Strategic Interventions

If coaches attempt to improve wrist-spin bowling performance, they first and foremost must be skilled enough to distinguish W1 from W2. Proficient coaches may even attempt to intervene and change a Type 2 wrist spinner into a predominantly Type 1 wrist spinner. To shift from a W2 to W1 delivery is not trivial: the bowling-arm plane, wrist-cocking method, and catch position may have to be changed to minimize precession during spin torque generation. In theory, precession would be minimized if the plane of arm motion during W2 spin torque generation were similar to that during W1 spin torque generation. Analogous changes in bowling-arm, wrist-cocking, and catch position will apply to finger spinners who wish to convert to a Type 1 technique. In general, a coach must have a sound understanding of spin bowling technique, as well as skills acquisition, to convert the actual mechanism of spin generation of wrist spinners and finger spinners in this way.

Alternatively, a wrist spinner may keep Type 2 wrist spin but use Type 1 finger spin as the main variation, the supination in both these deliveries used to disguise the change in direction of spin. The only caveat would be that the wrist spinner would need to modify the loading position of the Type 1 finger spinner so that it resembles the loading position of the wrist spinners. The wrist spinner can also learn both Type 1 and Type 2 wrist-spin deliveries, so that different forearm and finger motions produce the same spin direction [3]. This could serve as an effective means of deception, since batters usually associate different forearm and finger motions with different directions of spin, leading to an erroneous movement compensation that could lead to the batter’s dismissal. 

The highest-efficiency finger spinner is F1, which holds its corresponding slot in the performance ranking, which indicates it should be the preferred finger-spin delivery. However, there are differences in finger-spin types among populations, with Beach at al. [3] finding both F1 and F2 in their sample, whereas Sanders et al. [6] only found the pronation-type mechanism in their sample of finger spinners, most likely corresponding to the Type 2 finger-spin delivery. In essence, F2 should still be considered a feasible delivery based on its performance rating being above W2. Nevertheless, F1 and F2 should require distinct coaching instruction and partner different variations of deliveries. In Table 2, it can be observed that F1 can be paired with W2, since the forearm supinates in both deliveries (Figure 2), and that F2 can be paired with W1, which both require forearm pronation (Figure 2). Matching stock balls and variations using the same type of forearm rotation makes it more challenging for the batter to determine what type of variation was bowled. However, it is important to note that modified versions of W2 and W1 are used as variations with F1 and F2, respectively. The clearest example of this is the carrom ball [23], which is essentially W2 with a modified loading position. Correspondingly, the W1 variation that partners the F2 stock ball is also a modified delivery to make it resemble W1. 

### 4.3. Limitations and Future Studies

The major limitation of this study is that we had access to only one bowler, capable of bowling all four deliveries (F1, F2, W1, W2) at comparable performance. Nevertheless, the aim of this study was to explore whether the smart cricket ball can identify the differences between Type 1 and Type 2 spin, with the outlook to automatically detect these deliveries when feeding an artificial intelligence (AI) model with the smart ball data. The method developed in this study represents the first step in training an AI model. The recruitment of more bowlers, specifically bowlers coached for performing Type 2 spin deliveries, will be required for validating such an AI algorithm. 

## 5. Conclusions

In this study, it has been shown that the smart ball can differentiate between Type 1 and Type 2 wrist spin and finger spin on a range of physical performance and skill performance factors. These factors have implications for talent identification and performance enhancement. For instance, coaches can evaluate the efficiency of a spin bowler’s range of deliveries, i.e., repertoire. In addition, coaches can train to achieve difficult-to-decipher delivery sets based on the similarity of their release mechanics. Currently, the smart ball is the most effective way to measure these factors. Motion analysis does not seem to be a feasible option, because it would adversely impede bowing performance and consume excessive time and resources to test and evaluate the mechanics of the numerous finger segments during spin generation. When used by a knowledgeable coach, the smart ball can offer feedback on performance, suggest mechanical adjustments, and provide variation sets with more mechanical validity. In the future we aim to use the smart ball to analyse a greater range of deliveries, such as googlies, flippers, and swerve balls, providing a technical platform from which spin bowlers can improve their skill diversity and performance in an ever-changing cricket landscape.

## Figures and Tables

**Table 1:** Explanation of Type 1 and Type 2 deliveries.

Bowling Classification	Type	Finger Movement	Forearm Movement	Fingers and Forearm Move in the:
Finger spin (off spin)	Type 1	ulnar abduction	supination	**same** direction
Finger spin (off spin)	Type 2	ulnar abduction	pronation	**opposite** direction
Wrist spin (leg spin)	Type 1	radial abduction	pronation	**same** direction
Wrist spin (leg spin)	Type 2	radial abduction	supination	**opposite** direction

**Table 2:** Pairwise comparison of the 4 deliveries (F1 = finger spin type 1; F2 = finger spin type 2; W1 = wrist spin type 1; W2 = wrist spin type 2) resulting in 6 pairs for all 10 performance parameters; the common feature explains what the 2 deliveries of a specific pair have in common, according to Figure 2 (F = finger spin, W = wrist spin, T1 = type 1, T2 = type 2, P = pronation, S = supination); median 1 and 2 are the medians of the first and second delivery of each specific pair; U = unbiased estimator (statistic of Mann–Whitney test), p = probability (p-value), shown in bold font if p < 0.05; r = effect size (biserial correlation); effect = interpretation of r according to McGrath and Meyer [21], i.e., very small, small, medium or large (the term ‘zero’ indicates no effect at all, when U is at its maximum, and ‘separated’ means the data clusters do not overlap, when U = 0); the medians of the two deliveries of each pair are compared by means of a greater (>) sign or an equality (=) sign, depending on the p-value; the delivery with greater performance indicates which delivery out of one specific pair of deliveries shows the higher performance unless they have the same performance if p < 0.05 and thus are ‘equal’ (note that lower values are associated with higher performance for the following skill performance parameters: precession, normalised precession, precession torque, and *α*/*ω*).

Pairs	Common Feature	Median 1	Median 2	U	p	r	effect	Comparison of Medians	Delivery with Greater Performance
spin rate (angular velocity *ω*; revolutions per second)									
F1 F2	F	22.14	19.11	0	**0.0051**	1	separated	F1 > F2	F1
W1 W2	W	27.10	23.93	3	**0.0203**	0.833	large	W1 > W2	W1
F1 W1	T1	22.14	27.10	0	**0.0051**	1	separated	W1 > F1	W1
F2 W2	T2	19.11	23.93	0	**0.0051**	1	separated	W2 > F2	W2
F2 W1	P	19.11	27.10	0	**0.0051**	1	separated	W1 > F2	W1
F1 W2	S	22.14	23.93	8	0.1285	0.556	large	W2 = F1	equal
precession *p* (rad/s)									
F1 F2	F	27.11	32.63	0	**0.0051**	1	separated	F2 > F1	F1
W1 W2	W	9.19	19.43	0	**0.0051**	1	separated	W2 > W1	W1
F1 W1	T1	27.11	9.19	0	**0.0051**	1	separated	F1 > W1	W1
F2 W2	T2	32.63	19.43	0	**0.0051**	1	separated	F2 > W2	W2
F2 W1	P	32.63	9.19	0	**0.0051**	1	separated	F2 > W1	W1
F1 W2	S	27.11	19.43	0	**0.0051**	1	separated	F1 > W2	W2
normalised precession *p_n_* (°)									
F1 F2	F	54.62	58.78	10	0.2301	0.444	large	F2 = F1	equal
W1 W2	W	20.10	22.07	18	0.9362	0	zero	W1 = W2	equal
F1 W1	T1	54.62	20.10	0	**0.0051**	1	separated	F1 > W1	W1
F2 W2	T2	58.78	22.07	0	**0.0051**	1	separated	F2 > W2	W2
F2 W1	P	58.78	20.10	0	**0.0051**	1	separated	F2 > W1	W1
F1 W2	S	54.62	22.07	0	**0.0051**	1	separated	F1 > W2	W2
resultant torque *T_R_* (Nm)									
F1 F2	F	0.212	0.193	1	**0.0083**	0.944	large	F1 > F2	F1
W1 W2	W	0.227	0.216	11	0.2983	0.389	large	W1 = W2	equal
F1 W1	T1	0.212	0.227	11	0.2983	0.389	large	W1 = F1	equal
F2 W2	T2	0.193	0.216	11	0.2983	0.389	large	W2 = F2	equal
F2 W1	P	0.193	0.227	0	**0.0051**	1	separated	W1 > F2	W1
F1 W2	S	0.212	0.216	17	0.9362	0.056	very small	F1 = W2	equal
spin torque *T_s_* (Nm)									
F1 F2	F	0.195	0.175	1	**0.0083**	0.944	large	F1 > F2	F1
W1 W2	W	0.213	0.160	0	**0.0051**	1	separated	W1 > W2	W1
F1 W1	T1	0.195	0.213	5	**0.0455**	0.722	large	W1 > F1	W1
F2 W2	T2	0.175	0.160	3	**0.0203**	0.833	large	F2 > W2	F2
F2 W1	P	0.175	0.213	0	**0.0051**	1	separated	W1 > F2	W1
F1 W2	S	0.195	0.160	0	**0.0051**	1	separated	F1 > W2	F1
precession torque *T_p_* (Nm)									
F1 F2	F	0.107	0.099	5	**0.0455**	0.722	large	F1 > F2	F2
W1 W2	W	0.084	0.171	0	**0.0051**	1	separated	W2 > W1	W1
F1 W1	T1	0.107	0.084	6	0.0658	0.667	large	F1 = W1	equal
F2 W2	T2	0.099	0.171	0	**0.0051**	1	separated	W2 > F2	F2
F2 W1	P	0.099	0.084	8	0.1285	0.556	large	F2 = W1	equal
F1 W2	S	0.107	0.171	0	**0.0051**	1	separated	W2 > F1	F1
angular acceleration *α* (rad/s^2^)									
F1 F2	F	2508	2247	1	**0.0083**	0.944	large	F1 > F2	F1
W1 W2	W	2742	2055	0	**0.0051**	1	separated	W1 > W2	W1
F1 W1	T1	2508	2742	5	**0.0455**	0.722	large	W1 > F1	W1
F2 W2	T2	2247	2055	3	**0.0203**	0.833	large	F2 > W2	F2
F2 W1	P	2247	2742	0	**0.0051**	1	separated	W1 > F2	W1
F1 W2	S	2508	2055	0	**0.0051**	1	separated	F1 > W2	F1
power *P* (W)									
F1 F2	F	17.15	12.97	0	**0.0051**	1	separated	F1 > F2	F1
W1 W2	W	24.26	15.92	0	**0.0051**	1	separated	W1 > W2	W1
F1 W1	T1	17.15	24.26	0	**0.0051**	1	separated	W1 > F1	W1
F2 W2	T2	12.97	15.92	6	0.0658	0.667	large	W2 = F2	equal
F2 W1	P	12.97	24.26	0	**0.0051**	1	separated	W1 > F2	W1
F1 W2	S	17.15	15.92	13	0.4715	0.278	medium	F1 = W2	equal
efficiency *η* (%)									
F1 F2	F	64.64	64.43	17	0.9362	0.056	very small	F1 = F2	equal
W1 W2	W	88.32	65.21	0	**0.0051**	1	separated	W1 > W2	W1
F1 W1	T1	64.64	88.32	0	**0.0051**	1	separated	W1 > F1	W1
F2 W2	T2	64.43	65.21	16	0.8103	0.111	small	F2 = W2	equal
F2 W1	P	64.43	88.32	0	**0.0051**	1	separated	W1 > F2	W1
F1 W2	S	64.64	65.21	17	0.9362	0.0556	very small	F1 = W2	equal
*α*/*ω* (s^−1^; frequency, Hz)									
F1 F2	F	18.02	19.08	0	**0.0051**	1	separated	F2 > F1	F1
W1 W2	W	16.24	13.95	1	**0.0083**	0.944	large	W1 > W2	W2
F1 W1	T1	18.02	16.24	1	**0.0083**	0.944	large	F1 > W1	W1
F2 W2	T2	19.08	13.95	0	**0.0051**	1	separated	F2 > W2	W2
F2 W1	P	19.08	16.24	0	**0.0051**	1	separated	F2 > W1	W1
F1 W2	S	18.02	13.95	0	**0.0051**	1	separated	F1 > W2	W2

**Table 3:** Pairwise comparison of the 4 deliveries (F1 = finger spin type 1; F2 = finger spin type 2; W1 = wrist spin type 1; W2 = wrist spin type 2) resulting in 6 pairs for two performance parameter groups, that is, the combined physical and skill performance parameters; the combination was achieved by ranking each performance parameter across the 4 deliveries from highest (100%) to lowest (0%), and then combining these percentages of all physical performance parameters and all skill parameters for each delivery, resulting in 30 data per delivery and performance cohort; for further explanations cf. legend of Table 2.

Pairs	Common Feature	Median 1 (%)	Median 2 (%)	U	p	r	effect	Comparison of Medians	Delivery with Greater Performance
Physical Performance Parameters combined (physical performance order: W1 > F1 > F2 = W2)									
F1 F2	F	39.38	17.83	42	**<0.0001**	0.907	large	F1 > F2	F1
W1 W2	W	65.91	22.65	96	**<0.0001**	0.787	large	W1 > W2	W1
F1 W1	T1	39.38	65.91	75	**<0.0001**	0.833	large	W1 > F1	W1
F2 W2	T2	17.83	22.65	361	0.1902	0.198	small	F2 = W2	equal
F2 W1	P	17.83	65.91	2	**<0.0001**	0.996	large	W1 > F2	W1
F1 W2	S	39.38	22.65	252	**0.0035**	0.440	large	F1 > W2	F1
Skill Performance Parameters combined (skill performance order: W1 > W2 > F1 > F2)									
F1 F2	F	32.29	23.81	310	**0.0394**	0.311	medium	F1 > F2	F1
W1 W2	W	89.06	58.95	206	**0.0003**	0.542	large	W1 > W2	W1
F1 W1	T1	32.29	89.06	76	**<0.0001**	0.831	large	W1 > F1	W1
F2 W2	T2	23.81	58.95	232	**0.0013**	0.484	large	W2 > F2	W2
F2 W1	P	23.81	89.06	70	**<0.0001**	0.844	large	W1 > F2	W1
F1 W2	S	32.29	58.95	276	**0.0102**	0.387	large	W2 > F1	W2

**Table 4:** Correlations between two variables; R^2^ = coefficient of determination (**bold** font if R^2^ > 0.9); p = p-value (one-tailed); the data of the variables were taken at *t* = –0.44 s before the torque peak.

Variable 1 (Euler Angles of the Angular Velocity Vector)	Variable 2 (Components of the Unit Vector of the Angular Velocity)	R^2^	p
yaw	ω_x_	0.3953	0.0005
yaw	ω_y_	**0.9920**	<0.0001
yaw	ω_z_	0.0615	0.1207
pitch	ω_x_	0.2801	0.0040
pitch	ω_y_	0.0593	0.1255
pitch	ω_z_	**0.9938**	<0.0001

## Data Availability

The data presented in this study are available on request from the authors to any qualified researcher who has obtained Ethics Approval for secondary use of existing data through a Consent Waiver.
